# SAR-Constrained Wireless Power Transfer Modeling for an Implantable Optical Neurostimulator Sensors

**DOI:** 10.3390/s25237168

**Published:** 2025-11-24

**Authors:** So-Hyun Cho, Tahsin Nairuz, Jong-Ha Lee

**Affiliations:** Department of Biomedical Engineering, Keimyung University, Daegu 42601, Republic of Korea; 1114578@stu.kmu.ac.kr (S.-H.C.); tahsin.bmb@nstu.edu.bd (T.N.)

**Keywords:** multiphysics simulation, implantable optical device, wireless power transfer (WPT), specific absorption rate (SAR), optical absorption, neurostimulation

## Abstract

This study investigates the optimal operating conditions for an implantable photonic stimulation device, focusing on energy delivery efficiency and electromagnetic safety in biological tissue. COMSOL Multiphysics simulations were conducted to evaluate key light source parameters, including wavelength, output power, and incident angle. A transmitting RF coil was designed at a 1.35 MHz resonance frequency for wireless power transfer (WPT), and its resonant characteristics were analyzed using inductance and capacitance values. Specific Absorption Rate (SAR) simulations were performed with a 10 g hemispherical averaging region following international safety standards. Results showed that light absorption was maximized in the cerebellum and cerebrospinal fluid at a wavelength of 660 nm, with a 20° incident angle enabling the deepest tissue penetration. In vascular reflectance analysis, 660 nm wavelength produced the largest reflectance variation (∆R) across cardiac cycles and the lowest overall reflectance, indicating its suitability for optical biosignal detection and neural stimulation. SAR analysis demonstrated an average value of 0.0074 W/kg and a peak value of 0.82 W/kg, both substantially below the 2 W/kg safety threshold. These findings confirm that the proposed device design meets optical performance and biocompatibility requirements, highlighting its potential as a next-generation platform for precision phototherapy and future neurotherapeutic applications.

## 1. Introduction

Photobiomodulation therapy (PBMT) is a non-invasive therapeutic technique that uses near-infrared or red light to stimulate cellular metabolism, enhance mitochondrial activity, and promote tissue regeneration [1]. Recently, PBMT has attracted attention for its potential application in treating neurodegenerative diseases, such as Alzheimer’s disease [2,3,4]. In particular, previous studies have reported that PBMT applied to the hippocampus can induce the expression of brain-derived neurotrophic factor (BDNF), which may contribute to memory enhancement [5,6]. As a result, there have been growing efforts to develop technical strategies for effectively delivering light to the hippocampal region [7]. Several studies have explored implantable light-emitting devices and conducted computational simulations that incorporate the optical properties of biological tissues to improve light distribution and energy delivery [8,9].

Despite this progress, one of the significant challenges in translating PBMT into clinical practice remains the effective delivery of light to deep brain targets. Light in the visible and near-infrared spectrum undergoes substantial absorption and scattering as it propagates through biological tissue, which significantly limits penetration depth and reduces the efficiency of energy deposition in the intended regions. To address these limitations, previous research has focused on identifying the optimal irradiation parameters—including wavelength, power, and incidence angle—that maximize optical energy delivery to deep brain regions [8,10]. By modeling brain tissue as a multilayered structure with distinct optical properties, researchers have quantitatively analyzed light absorption and penetration depth across a range of wavelengths. These simulation-based investigations consistently indicate that a wavelength of approximately 660 nm combined with a 20° incidence angle achieves superior energy deposition in the hippocampal region while maintaining a notably low thermal effect, thereby ensuring favorable safety conditions. Furthermore, mesh independence tests and evaluations of absorption and scattering coefficients have been performed to validate the accuracy and reliability of these simulation models [10].

However, most existing models of light propagation are based on static tissue conditions and do not account for dynamic physiological changes such as blood flow or oxygenation [11]. Blood is a critical component that significantly influences light transport in biological tissues. In the human body, blood flow varies over time due to cardiac cycles, and the oxygenation state of blood alters its optical absorption properties. Oxyhemoglobin (HbO_2_) and deoxyhemoglobin (Hb) exhibit distinct absorption characteristics depending on the wavelength [12]. For instance, HbO_2_ shows high absorption between 540–580 nm, while Hb exhibits a strong absorption peak near 760 nm. Neglecting these spectral differences may lead to inaccurate predictions regarding the effectiveness and safety of photonic stimulation [13]. Jacques (2013) emphasized the importance of treating blood flow and hemoglobin state as variables in light transport modeling, as they affect wavelength-specific absorption behavior [14]. According to open datasets provided by the OMLC, the absorption coefficient of HbO_2_ and Hb can differ by more than a factor of 10 within the 400–900 nm range [15]. Simulations based on these data allow for the quantification of changes in light penetration and absorption patterns depending on the presence of blood flow or variations in oxygen saturation.

At the same time, as PBMT technologies move toward higher output levels and evolve into implantable or skin-contact systems, evaluating electromagnetic exposure within tissues has become increasingly important [16]. Implantable systems using wireless power transfer (WPT) eliminate physical connectors and reduce infection risks, but they also generate localized electromagnetic fields that can lead to tissue heating or excessive energy absorption. To address these risks, international standards, such as IEEE (C95.3-2002) and the International Commission on Non-Ionizing Radiation Protection (ICNIRP), define safety thresholds based on the specific absorption rate (SAR), which quantifies the absorption of electromagnetic energy in biological tissue [17]. Regulatory thresholds generally require SAR values to remain below 1.6 W/kg over 1 g of tissue or 2.0 W/kg over 10 g, depending on the exposure conditions [18]. Despite significant progress in optimizing light penetration and output in PBMT devices, relatively few studies have thoroughly evaluated SAR or addressed electromagnetic safety considerations. This is particularly important in implantable configurations, where the device operates in direct contact with neural or subcutaneous tissue, exposing the surrounding region to localized electromagnetic fields generated by the applied current [19]. Under such conditions, it is essential to predict SAR levels quantitatively and verify compliance with international safety standards. Failure to meet these standards may result in local heat accumulation or tissue damage [20]. Consequently, SAR modeling and electromagnetic exposure simulations have emerged as essential components of safe and effective implantable neurostimulation device design.

Szlawski et al. calculated SAR values in brain tissue based on electromagnetic fields generated during the operation of an inductively coupled modular brain–machine interface device [21]. Similarly, modeling studies have predicted temperature increases and SAR (or energy-deposition) distributions during optical/infrared neural stimulation: earlier finite-element models characterized the temporal and spatial heating produced by pulse trains and examined dependence on pulse length, repetition rate and irradiation geometry, while more recent work combined numerical simulations with MR thermometry to quantify temperature directly rise as a function of radiant exposure, fiber size and stimulus protocol [22]. Both modeling and experimental studies integrated thermal conductivity and tissue density (in addition to electrical/optical properties) to evaluate the safety/efficacy trade-offs. Homann et al. proposed a methodology for patient-specific SAR modeling using Finite-Difference Time-Domain (FDTD) simulations. Their models were compared with B1^+^ field maps acquired from MRI scans, which visualize the distribution of RF energy in tissue. The correlation coefficient between simulated and measured values reached up to 71%, confirming that the model accurately reflected in vivo electromagnetic propagation [23]. In addition to these approaches, researchers from Tbilisi State University have applied the Method of Auxiliary Sources (MAS) to SAR dosimetry, with Jeladze et al. (2025) reporting accurate numerical estimation of SAR distribution in biological organisms exposed to far-field RF fields, underscoring the relevance of MAS for modeling heterogeneous tissue structures [24]. All these studies highlight the necessity of robust numerical techniques for evaluating electromagnetic exposure and ensuring the safety and efficacy of emerging biomedical devices.

Based on these findings, the present study builds upon previously proposed implantable light source designs and optimizes irradiation parameters to enhance the precision of light energy delivery in vivo. Particular attention was given to the fact that light absorption and scattering can vary depending on the oxygenation state of blood. To account for this, vascular structures were incorporated into the tissue model, and wavelength-dependent absorption and scattering coefficients of HbO_2_ and Hb were applied. This analysis was used to determine whether light penetration depth and energy distribution could be preserved under fixed output conditions. In addition, the electromagnetic safety of brain tissue and surrounding soft tissues was quantitatively assessed using the 1 *g* SAR criterion. This allowed us to evaluate whether therapeutic light could be delivered effectively without exceeding safety thresholds.

Ultimately, this study presents a simulation framework that integrates oxygenation-dependent light absorption modeling with SAR-based electromagnetic safety analysis. This combined approach enables simultaneous verification of both the efficacy and biocompatibility of implantable PBMT devices. It addresses limitations of previous static models and provides a scientific foundation for device design in future clinical applications. The results may contribute to the establishment of technical guidelines for personalized neurotherapeutic strategies and smart bio-integrated systems.

## 2. Research Subjects and Method

In this study, simulations were conducted using COMSOL Multiphysics 6.0 to simultaneously account for the optical properties of blood flow and the electromagnetic response of biological tissues. The simulation approach was divided into two main components. First, a two-dimensional wave optics model was implemented to analyze the detailed characteristics of light propagation through tissue. Second, a three-dimensional SAR analysis model was constructed to evaluate the spatial distribution of absorbed electromagnetic energy and to assess safety within the biological environment.

### 2.1. Modeling

The optical simulation model incorporating blood flow consisted of four light sources, each with a diameter of 0.3 mm. The incident angle was fixed at 20°, as determined in our prior analysis [10]. In that previous study, a dedicated angular optimization demonstrated that a 20° incidence provided the greatest penetration depth and highest energy deposition in deep brain tissues. To maintain methodological consistency and because the focus of the present work is on integrating vascular optical behavior with SAR evaluation rather than reoptimizing angular parameters, the previously validated 20° angle was adopted as a fixed input. The power of each light source was set to 3 mW. For each wavelength and tissue composition, light penetration depth, residual energy, and electric field distribution were quantitatively evaluated. Here, the electric field refers to the internally induced electromagnetic response caused by photon-induced charge density fluctuations, rather than externally applied current. This electric field pattern reflected the actual propagation path of the light energy.

**Figure 1:**
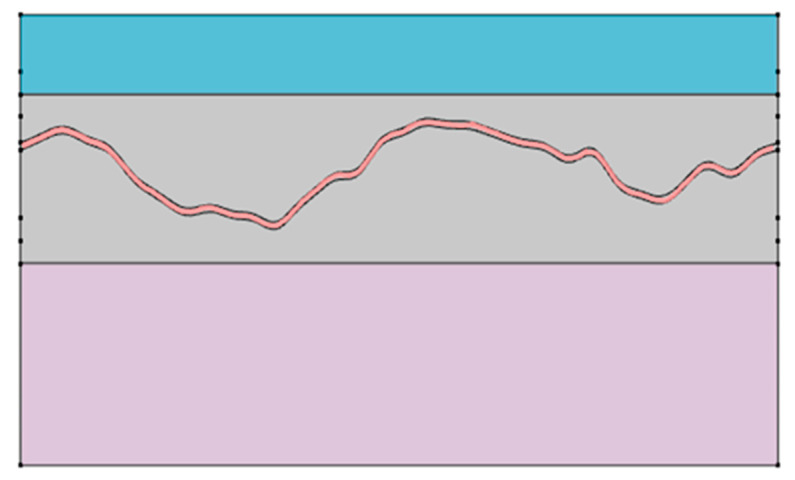
Two-dimensional optical simulation model incorporating curved brain anatomy and vascular structure. The x-axis represents the horizontal spatial coordinate (mm), and the y-axis represents the vertical spatial coordinate (mm). The model includes cerebrospinal fluid, gray matter, cerebellum, and an embedded blood vessel, enabling wavelength-dependent analysis of light propagation and reflectance under dynamic vascular conditions.

As shown in Figure 1, the vascular simulation incorporated curved anatomical structures such as cerebrospinal fluid, gray matter, and the cerebellum. A vessel was embedded within the gray matter. To ensure computational efficiency and reproducibility, the model was implemented in two dimensions. This configuration enabled rapid assessment of energy transfer patterns under varying wavelengths.

**Figure 2:**
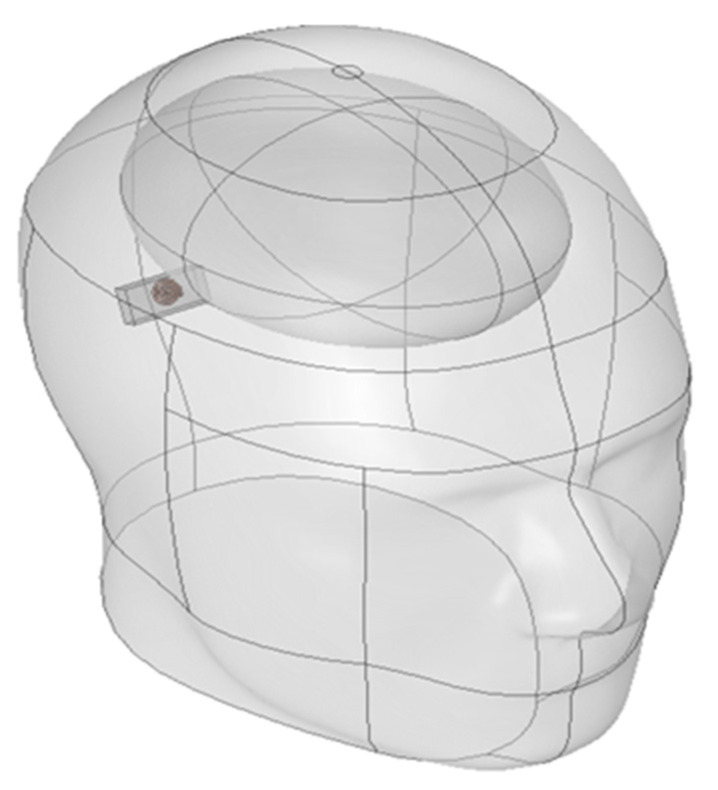
Three-dimensional SAR simulation model of the head with implantable device for electromagnetic safety evaluation.

The SAR analysis for evaluating biological safety was conducted using a separate three-dimensional electromagnetic model, as illustrated in Figure 2. This model included the skull, brain, and the implantable device. The analysis focused on quantifying the electromagnetic energy absorbed per unit volume. For accurate SAR calculation, the simulation incorporated the electrical conductivity, relative permittivity, and density of each tissue. These parameters are crucial for evaluating power limits and assessing potential damage risk in accordance with international safety guidelines [25]. Furthermore, a comparison between the absorbed energy in the brain and the entire head was performed to confirm safety with respect to local energy accumulation.

### 2.2. Blood Pressure Simulation

This simulation aimed to provide fundamental data for the development of light-based blood flow detection and therapeutic technologies, as well as to improve the precision of light transport modeling in biological tissues. A tissue model incorporating blood vessels and surrounding anatomical structures was constructed to simulate the reflection spectrum.

Reflectance variation (∆R) was used as the key analysis metric, representing the relative change in reflectance as a function of wavelength. The ∆R values were calculated based on the absorption coefficients of Hb and HbO_2_ across the spectral range. This allowed for quantitative evaluation of how changes in blood flow and oxygen saturation levels influence optical reflectance characteristics [26,27].

### 2.3. Optical Properties of Modeling

The theoretical absorption and scattering coefficients used in the simulations were based on the data presented in Table 1 and Table 2. Table 3 summarizes the physical parameters and governing equations used in the SAR analysis. These values served as a foundation for evaluating the amount of electromagnetic energy absorbed by each tissue component under varying power levels. In addition to optical properties, thermal characteristics were also incorporated into each simulation to account for potential heat generation. Specifically, the specific heat capacity was used as a key indicator for determining how quickly absorbed photonic energy is converted into temperature rise within tissue. All property values were referenced from previous studies and literature on biological tissues [14,21,28,29,30,31].

### 2.4. Blood Pressure Simlation Method

Simulation results were obtained using two complementary approaches to ensure the accuracy of the wavelength-dependent reflectance model. First, the theoretical reflectance variation, ΔR_J, was derived from the theoretical absorption coefficients of Hb and HbO_2_ reported in previous literature [26,27] using Beer–Lambert–based reflectance calculations. For each wavelength, theoretical reflectance was estimated from these coefficients, and ΔR_J was defined as the difference between the maximum and minimum reflectance values within the analyzed spectral band. Second, the simulation-based reflectance variation, ΔR_simulation, was obtained directly from the COMSOL optical model by computing the reflected light intensity at each wavelength while incorporating vascular geometry, tissue optical properties, and time-varying blood pressure changes. The difference between the highest and lowest simulated reflectance values was defined as ΔR_simulation. These two parameters enabled cross-validation between theoretical predictions and numerical simulations.

**Figure 3:**
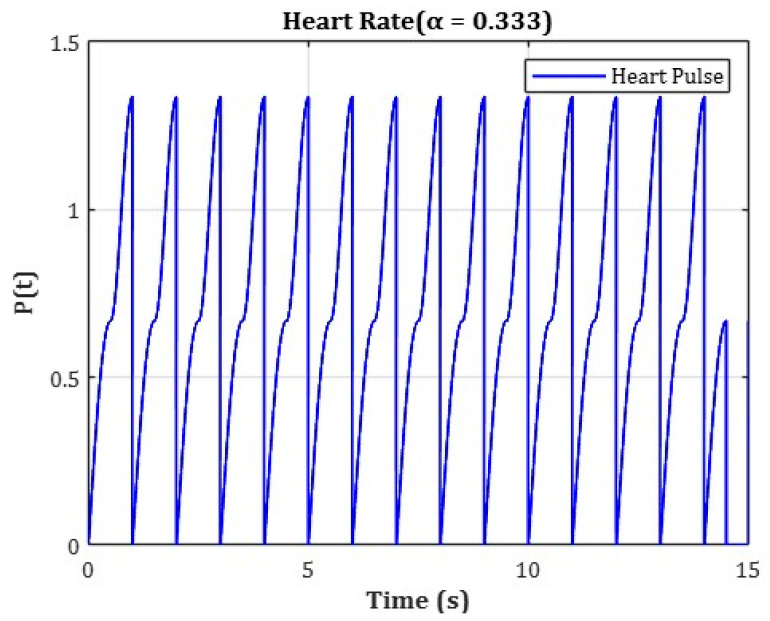
Time-dependent blood pressure waveform applied to vascular model to simulate cardiac-induced hemodynamic variations.

To analyze wavelength-specific behavior, the analysis was conducted by dividing the spectral range into three distinct wavelength bands: (1) 400–500 nm, (2) 500–600 nm, and (3) 600–800 nm. For each range, the change in ∆R was analyzed independently. To more accurately reflect real-time hemodynamic variations, the numerical model incorporated cardiac-cycle–induced blood-pressure variations, as illustrated in Figure 3.

The cardiac cycle was set to a total duration of 1.5 s, and pressure variation over time was applied using separate functions for each phase. The initial phase (0–0.5 s) represented an artificial ramp-up interval used solely for numerical convergence and initialization purposes, without any physiological significance. During this initial ramp-up phase, blood pressure variation was defined using the following function:

(1) $$P(t)=(1-\alpha )\cdot sin(\pi \left(t-tstart\right))$$

The physiologically relevant cardiac phase was set from 0.5 s to 1.5 s, representing a 1.0 s interval. During this period, the blood pressure was modeled as a periodic oscillation between minimum and maximum values using a triangular waveform function. This function was designed based on the average adult heart rate of approximately 75 beats per minute (bpm), where one cardiac cycle lasts about 0.8 s. To ensure numerical stability, the time window was extended beyond a single heartbeat for simulation purposes. The following function was applied during the main pulsation phase:

(2) $$P(t)=(1-\alpha )\cdot cos(2\pi \left(t-tstart\right))$$

In this model, α is a coefficient set to reflect the physiological ratio of the human cardiac cycle, where systole occupies approximately 33.3% of the total duration.

The variable t represents time (in seconds), and tstart indicates the starting time of each phase. This function was designed to realistically simulate pressure variations over a cardiac cycle by incorporating the ratio between systolic and diastolic phases.

By integrating such time-dependent blood pressure changes, the simulation enables reflectance analysis under dynamic circulatory conditions rather than in a static environment. This approach to temporal segmentation has also been adopted in the official vascular models provided by COMSOL Multiphysics. It ensures physiological accuracy in pulsation modeling while maintaining high temporal resolution and simple function definitions [32].

### 2.5. SAR Simulation Modeling

**Figure 4:**
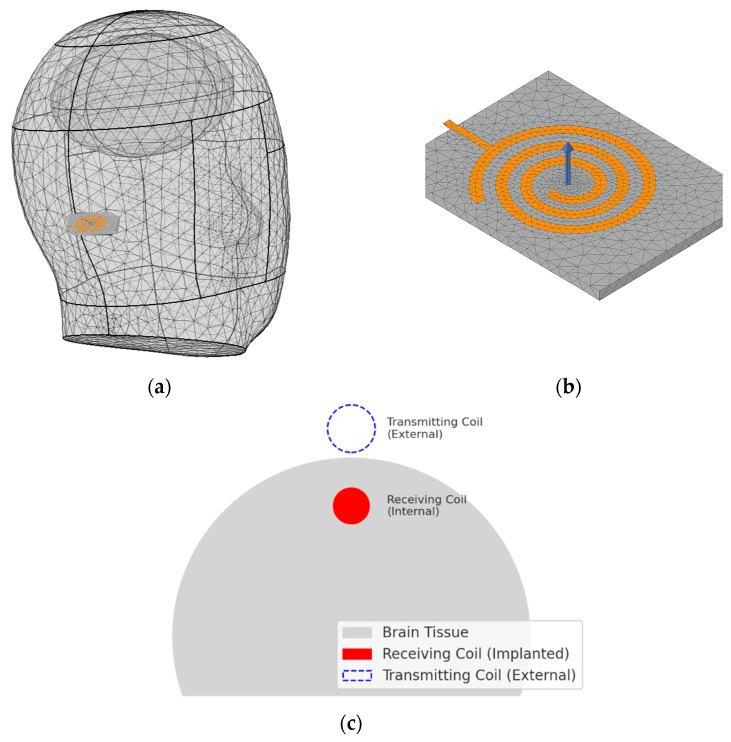
SAR simulation model with a 1.35 MHz WPT coil embedded in an implantable device: (**a**) Three-dimensional anatomical modeling of head and implant region, (**b**) Coil geometry and structural configuration, (**c**) Overall system schematic illustrating the resonant inductive coupling architecture between the external transmitting coil and the implanted receiving coil for wireless power delivery.

In this study, WPT system was implemented using a resonant inductive coupling architecture, consisting of an external transmitting coil and an implantable receiving coil. The receiving coil embedded in the device was designed with an inductance of approximately 15 µH, and a 100 pF parallel capacitor was added to form a compact LC resonant circuit. The resonance frequency is a critical design parameter in WPT systems because it directly affects power-transfer efficiency and the effective distribution of electromagnetic fields within biological tissue [33]. As shown in Figure 4, the target resonance frequency of the coil embedded within the device was set to 1.35 MHz.

Theoretically, the resonance frequency can be calculated using the LC resonance equation.

(3) $$f_{0}=\frac{1}{2\pi \sqrt{LC}}$$

where L represents the inductance of the coil and C denotes the capacitance of the parallel capacitor. In this study, a coil with an inductance of approximately 15 μH was assumed. The system was designed to resonate at 1.35 MHz, and the required capacitance was back-calculated accordingly. Based on this calculation, a parallel capacitor with a capacitance of approximately 100 pF satisfies the resonance condition.

To verify the validity of the 1.35 MHz resonance setting, the $S_{11}\;$(Return Loss) characteristic of the coil was simulated. $S_{11}$ is the logarithmic measure of the reflection coefficient and is defined by the following relationship:

(4) $$Return\;Loss\left(dB\right)=-20\cdot {log}_{10}(|S_{11}|)$$

**Figure 5:**
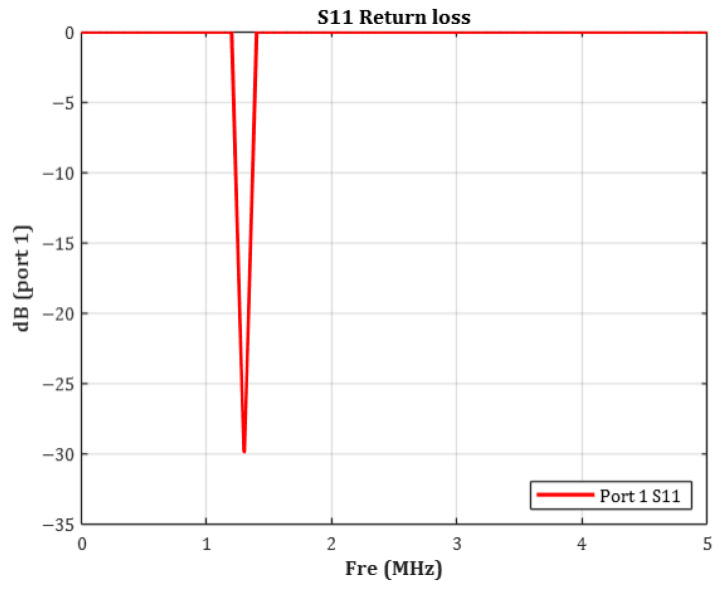
Simulated return loss ($S_{11}$) curve of the WPT coil, showing resonance at 1.35 MHz with measured S_11_ = −29.87 dB.

In general, an S_11_ value below −10 dB indicates acceptable impedance matching. Values below −20 dB signify that less than 1% of the input power is reflected, representing excellent matching [34]. As shown in Figure 5, the measured $S_{11}$ was −29.87 dB, corresponding to an absolute reflection coefficient of approximately 0.032. This indicates that over 95% of the input power was delivered to the coil with minimal loss. These results confirm that the simulated resonance behavior closely aligns with the theoretical target frequency.

Thus, the LC resonance design based on the calculated inductance and capacitance was effectively implemented, validating the system’s successful operation at 1.35 MHz.

### 2.6. SAR Simulation Method

In this study, the resonance frequency of the transmitting coil was set to 1.35 MHz. The $S_{11}$ return loss simulation confirmed a resonance peak at −29.87 dB, indicating excellent impedance matching at the transmitter and minimal power reflection at the target frequency.

Based on this result, 1.35 MHz was validated as an effective operating frequency for wireless power transfer. Subsequently, SAR simulations were performed using a hemispherical human tissue model to evaluate electromagnetic energy absorption at this frequency. The goal was to determine whether the amount of energy absorbed by biological tissues under the specified conditions remained within regulatory safety limits. Importantly, the analysis focused not on the electric field distribution generated by the transmitter (external charger), but on the electromagnetic field induced directly by the implanted receiving device. This approach followed a bio-applicable model designed to reflect actual implant usage conditions.

SAR was calculated using the following equation, which quantifies the rate at which energy is absorbed by tissue under electromagnetic exposure:

(5) $$SAR=\frac{\sigma \cdot |{E|}^{2}}{\rho }$$

In the SAR equation, σ represents the electrical conductivity of tissue (S/m). This parameter indicates how easily electrical current can flow through the tissue when exposed to electromagnetic waves and is closely related to the rate at which electromagnetic energy is converted into heat. The variable E denotes the electric field strength (V/m), representing the intensity of the electromagnetic field formed within tissue during device operation, such as optical activation or wireless charging. SAR is proportional to the square of E, making it a dominant factor in determining energy absorption. The parameter ρ corresponds to the tissue density ($kg/m^{3}$), which reflects the mass per unit volume. Since SAR expresses the energy absorbed per unit mass, ρ acts as a normalization factor in the overall calculation.

In this study, SAR simulation was conducted in accordance with international electromagnetic exposure safety standards, including those from ICNIRP and IEEE. Specifically, the 10 g average SAR (SAR_10_g) method was adopted, in which the mean electromagnetic energy absorbed within a 10 g mass of tissue is calculated to ensure biocompatibility and safety compliance [35,36].

In SAR analysis, the averaging region is a critical component used to quantify the localized absorption of electromagnetic energy within biological tissues. This region is typically modeled as a spherical or hemispherical domain, based on the assumption that electromagnetic fields diffuse isotropically within tissue, spreading uniformly in all directions without directional bias. As such, a spherical domain is considered the most appropriate structure to capture the symmetry of energy distribution. In contrast, if the averaging domain is defined as a sharp-edged cuboid, it may introduce electric field discontinuities or distorted absorption values near the boundaries. Therefore, spherical or hemispherical domains provide more numerically stable conditions for SAR evaluation. Based on both physical reasoning and numerical considerations, a spherical averaging model was initially selected. However, since the implantable device in this study was placed adjacent to the skull surface, a hemispherical domain was ultimately adopted for the actual simulation. This configuration better reflects the anatomical constraint of the implantation site.

The method for calculating the hemispherical averaging volume is as follows. The average tissue density (ρ) was set to 1007 $kg/m^{3}$. Based on this value, the volume corresponding to an idealized 10 g (0.01 kg) tissue mass was computed using the following formula:

(6) $$V=\frac{m}{\rho }=\frac{0.01\;kg}{1007\;{kg/m}^{3}}\approx 9.9\times 10^{-6\;}m^{3}$$

Based on this volume, the radius of the sphere is inversely calculated

(7) $$r={\left(\frac{3V}{4\pi }\right)}^{1/3}\approx 1.33\;cm$$

It can be seen that a spherical region with a radius of about 1.33 cm corresponds to 10 g of biological tissue.

However, in the actual simulation setup, the implantable device is positioned adjacent to the brain surface. Under this condition, a full spherical averaging domain may partially extend into the surrounding air region. Since air does not absorb electromagnetic energy and is not considered in SAR calculations, the inclusion of air within the averaging domain could artificially lower the SAR value. This would lead to a distorted evaluation of tissue energy absorption. To address this issue, a hemispherical averaging domain was applied instead of a full sphere. This approach better aligns with the anatomical placement of the device, ensuring that only biologically relevant tissue regions are included in the SAR computation.

**Figure 6:**
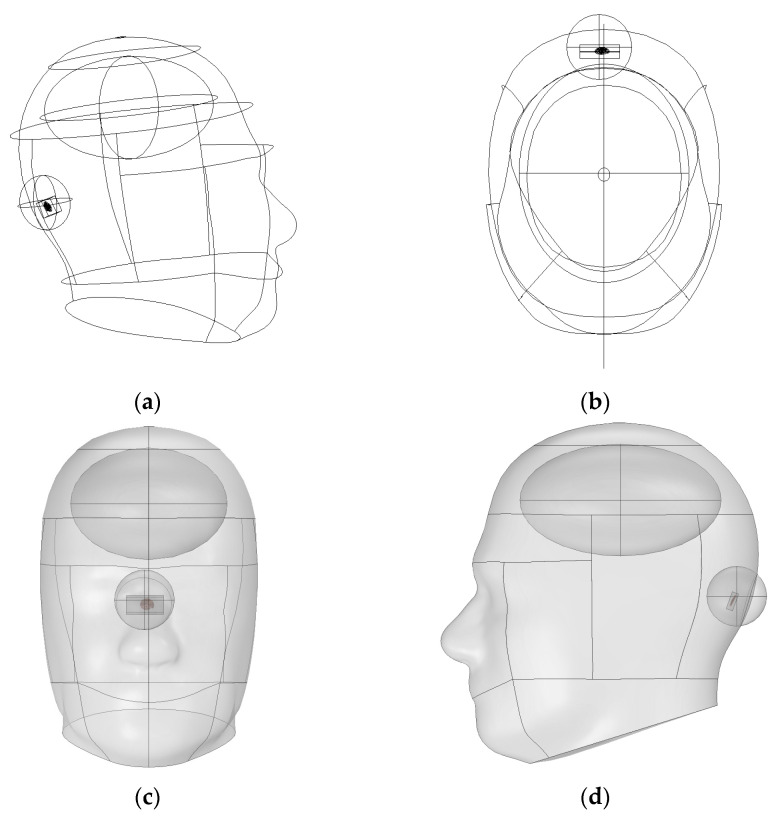
Geometry of the SAR averaging domain using a hemispherical model: (**a**) Side view of the line-based cross-section, (**b**) top view showing hemispherical boundary alignment, (**c**) front view of the surface geometry, (**d**) detailed side view of the full brain-device configuration.

As illustrated in Figure 6, the averaging region was strictly confined to intracranial tissue, thereby preventing underestimation and maintaining accuracy in SAR evaluation. Moreover, the adoption of spherical or hemispherical domains offers several scientific advantages. First, electromagnetic waves do not propagate linearly in biological media. Due to refraction and scattering, they spread spatially in multiple directions.

As a result, curved-surface domains provide boundary conditions that more accurately reflect the directional nature of wave propagation. Second, curved boundaries minimize edge reflections and scattering, and better capture the isotropic distribution of electromagnetic energy within tissue. This enables more realistic modeling of field behavior in complex biological environments. Third, such averaging schemes reduce boundary-related numerical artifacts and field concentration effects. This leads to enhanced simulation accuracy and repeatability, particularly in regions near the implantable device where tissue heterogeneity and irregular boundaries are present. Compared to cuboid-based averaging cells, hemispherical mass-averaging provides more reliable and precise outcomes in anatomically realistic simulations.

## 3. Experimental Results

### 3.1. Vessel Simulation

**Figure 7:**
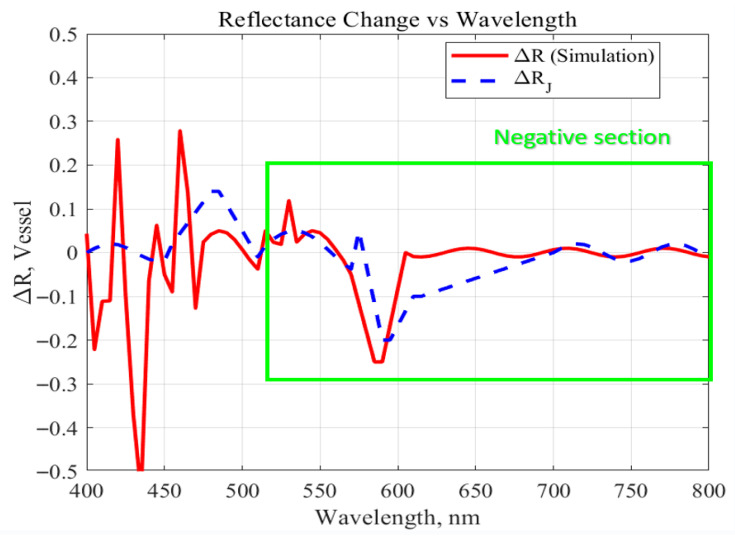
Comparison of simulated and theoretical reflectance variation (∆R).

The simulation results showed that the variation in vascular reflectance (∆R) exhibited distinct patterns across three spectral ranges. As shown in Figure 7, the analysis was conducted by comparing ∆R_simulation, obtained from the numerical model, with ∆R_J, which was calculated using theoretical absorption coefficients.

(1) In the 400–500 nm range, ∆R showed large fluctuations in amplitude. This region corresponds to the strong absorption band of both Hb and HbO_2_, where significant light absorption occurs within blood, resulting in pronounced reflectance changes.

(2) In the 500–600 nm range, the overall reflectance gradually decreased. This trend was attributed to the sustained absorption by hemoglobin, which led to cumulative attenuation of light through the tissue. Both ∆R_simulation and ΔR_J showed a similar declining pattern, although the simulation exhibited slightly greater reduction due to its incorporation of dynamic blood flow conditions.

(3) Above 600 nm, reflectance began to increase gradually. This region is characterized by weaker absorption by hemoglobin, allowing light to penetrate the blood more effectively and reach deeper tissue layers. During conditions of vasodilation or increased blood flow, a slight increase in reflectance was observed; however, overall reflectance remained stable and exhibited a recovery trend. The difference between ∆R_simulation and ∆R_J was relatively small in this spectral region, indicating that the simulation accurately reproduced the wavelength-dependent optical reflectance behavior of hemoglobin under dynamic physiological conditions.

While Figure 7 illustrates the overall spectral trends, the identification of 660 nm as the optimal wavelength was determined from numerical analysis of both ΔR_simulation and ΔR_J. Within the post-600 nm recovery region—where hemoglobin absorption weakens and tissue penetration improves—the numerical data showed that 660 nm produced the largest pulsatile reflectance variation (highest ΔR) and the lowest baseline reflectance. This combination indicates that more optical energy is absorbed by the tissue while still remaining strongly modulated by changes in blood volume during the cardiac cycle, demonstrating high sensitivity to hemodynamic fluctuations. These characteristics show that 660 nm achieves an optimal balance between optical absorption, penetration depth, and responsiveness to blood flow dynamics. Based on this combined theoretical and simulation evidence, 660 nm was selected as the reference wavelength for subsequent optical and SAR analyses in this study.

### 3.2. SAR Simulation

The implantable photonic stimulation device designed in this study receives power wirelessly at a resonance frequency of 1.35 MHz. To evaluate the potential accumulation of electromagnetic energy in brain tissue during wireless charging, SAR analysis was conducted to assess electromagnetic biocompatibility. The SAR calculation was based on the electric field generated within the photodiode, corresponding to the actual operational condition of the implanted device.

**Figure 8:**
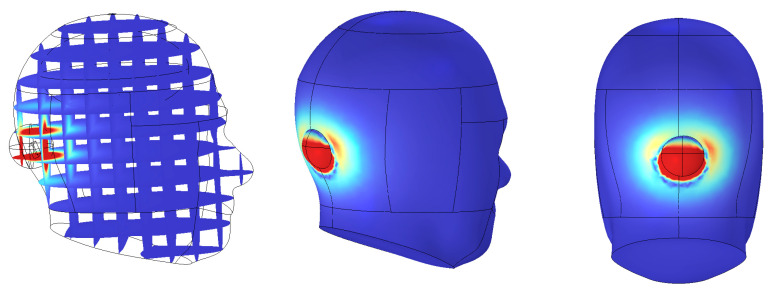
Three-dimensional visualization of SAR distribution within spherical averaging domain at 1.35 MHz.

The simulation was performed under optimized optical parameters: a wavelength of 660 nm, power output of 3 mW, and an incident angle of 20°. As illustrated in Figure 8, the electromagnetic energy absorption within the spherical domain was visualized in three-dimensional volume form.

According to Table 4, the average SAR was calculated to be approximately 0.0074 W/kg, with a peak SAR value of 0.8252 W/kg. These values are significantly lower than the 2 W/kg limit set by the ICNIRP guidelines for localized exposure. The results confirm that the electromagnetic field induced by the device during wireless charging does not pose a thermal or physiological risk to brain tissue.

**Figure 9:**
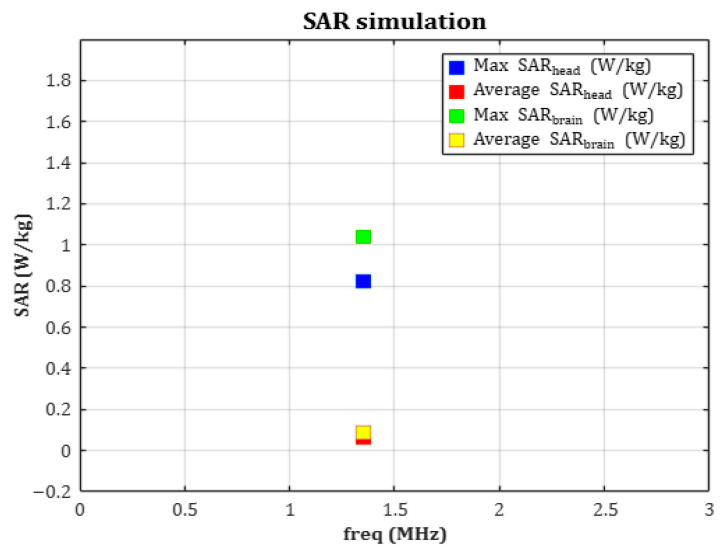
Comparison of maximum and average SAR values for the brain and entire head, based on hemispherical averaging.

Figure 9 presents a graphical comparison of the maximum and average SAR values for both the brain and the entire head, calculated using the hemispherical averaging domain. All four metrics are clearly below the international safety limits, demonstrating the electromagnetic safety of the system.

## 4. Discussion

This study presents an integrated simulation framework that simultaneously evaluates optical efficacy and electromagnetic safety for implantable photonic neurostimulation devices. Although optical absorption modeling and SAR evaluation represent independent physical domains, they define two essential and complementary constraints on the same implantable system. For an implanted device to be clinically viable, it must deliver sufficient optical energy to deep-brain targets while ensuring that wireless power transfer does not expose surrounding tissue to unsafe electromagnetic fields. By combining these analyses in a unified methodology, the present work addresses a critical gap in existing research, where optical and electromagnetic design considerations are often treated separately.

Using COMSOL-based multiphysics simulations, this study systematically analyzed combinations of key optical parameters—wavelength, power, and incident angle—to determine their influence on penetration depth, vascular sensitivity, and energy deposition within brain tissue. Across the 400–800 nm range, incorporating the absorption characteristics of HbO_2_ and Hb, revealed that 660 nm consistently demonstrated the most favorable optical behavior. Numerical evaluation of both theoretical (ΔR_J) and simulated (ΔR_simulation) reflectance showed that 660 nm produced the largest pulsatile reflectance variation and the lowest baseline reflectance, indicating superior sensitivity to hemodynamic modulation during the cardiac cycle. In addition, this wavelength achieved deep penetration and strong absorption in critical brain regions such as the cerebrospinal fluid and cerebellum while maintaining relatively low scattering. These characteristics align with established hemoglobin absorption trends in the visible–near-infrared window [12,15] and confirm 660 nm as an effective wavelength for both photobiomodulation and blood-flow-sensitive biosignal detection. Furthermore, an incident angle of 20° yielded the greatest tissue penetration depth and energy absorption, identifying it as an essential design parameter for optimizing photodiode or light-source placement in implantable photonic devices.

In parallel, SAR analysis was performed to evaluate the electromagnetic energy absorbed by human tissue during wireless power transfer. The receiving coil, resonating at 1.35 MHz, generated average and peak SAR values of approximately 0.0074 W/kg and 0.8255 W/kg, respectively—both far below the ICNIRP-recommended limit of 2 W/kg. By applying a hemispherical 10 g averaging model, both the accuracy of the analysis and biological safety were ensured. Mesh independence validation was also conducted, confirming the numerical stability and reproducibility of the simulation. The choice of a 1.35 MHz resonance frequency was driven by both electromagnetic safety and device-integration considerations for implantable WPT systems. Operating in the low-MHz magneto-quasistatic regime minimizes electric-field absorption in biological tissues, enabling efficient power transfer while maintaining SAR well within international safety thresholds. This frequency selection is consistent with prior studies demonstrating that 1–15 MHz provides strong magnetic coupling and minimal tissue heating in implantable biomedical devices [33,37]. Additionally, for the receiving coil used in this device (≈15 µH), 1.35 MHz satisfies the LC resonance condition with a compact parallel capacitor (~100 pF), supporting stable resonance and practical device integration. Together, these results validate 1.35 MHz as an effective and safe operating frequency for the proposed implantable WPT system.

In SAR analysis, a fixed coil configuration was employed to evaluate electromagnetic exposure under the device’s intended operational condition. Although the distance, relative position, and angular misalignment between the transmitting and receiving coils are known to influence WPT efficiency and SAR distribution, these variables were held constant in this study. The receiving coil embedded within the implant was directly excited at its resonant frequency to model induced fields in biological tissue, while the transmitting coil was not treated as a variable element. This approach is consistent with standard safety assessment practices for implantable biomedical systems, which prioritize evaluation of localized field exposure rather than user-dependent charging variability. Nevertheless, future work will extend this analysis by systematically examining the effects of coil separation, positional offsets, and angular misalignment on both SAR and WPT efficiency. Integrating these evaluations with the optical–electromagnetic modeling framework presented here will provide a more complete understanding of power-delivery performance and support the development of fully validated, clinically deployable implantable photonic systems.

Nonetheless, the significance of this study lies in establishing a unified and comprehensive framework that validates the optical efficiency, thermal safety, and electromagnetic compatibility of an implantable phototherapy device. Although optical absorption and SAR arise from different physical mechanisms and do not directly influence one another, they impose simultaneous and interdependent design constraints on the same implant platform. By demonstrating that an optically optimized configuration—660 nm wavelength, 20° incidence angle, and 3 mW power—also satisfies electromagnetic safety requirements during resonant inductive wireless powering, this work provides a multidimensional and realistic validation strategy for preclinical device evaluation. This integrated approach addresses a key challenge faced by emerging bio-integrated light-delivery systems [8,9], which must balance optical penetration, hemodynamic sensitivity, power-delivery efficiency, and thermal considerations. The proposed framework therefore expands the feasible design space for next-generation implantable photonic devices, enabling researchers to verify that a selected optical configuration will not compromise electromagnetic biocompatibility. The findings further establish a scientific foundation for the development of personalized phototherapeutic devices, neuromodulation tools, and smart biosensor systems. Ultimately, this combined methodology supports the creation of safe, effective, and clinically translatable light–electronics fusion therapies for precision medicine.

Although this study offers a comprehensive evaluation of optical behavior and electromagnetic safety, several limitations should be acknowledged. First, the wireless power transfer (WPT) analysis was conducted at the electromagnetic field level, focusing primarily on SAR assessment. As a result, key electrical parameters—including the output voltage and current delivered to the photonic module, the coil-to-coil coupling coefficient, and the overall transmission efficiency—were not modeled. These depend on coil alignment, separation distance, tissue loading, and rectifier circuitry, which were beyond the scope of the present safety-focused framework. Second, while the optical and electromagnetic simulations were extensive, they have not yet been validated experimentally. Biological complexities, such as heterogeneous tissue composition, anisotropic scattering, and metabolic variations, may introduce deviations from numerical predictions. Third, the optical model was implemented in two dimensions for computational tractability, which may underestimate scattering and absorption effects in anatomically complex three-dimensional regions. Furthermore, only a single coil geometry and resonance frequency were evaluated, potentially limiting the generalizability of the WPT findings.

Future work should incorporate full circuit-level modeling to quantify coupling efficiency, voltage/current delivery, and link reliability under realistic implantation conditions. Three-dimensional optical modeling should also be performed to better capture light–tissue interactions in complex anatomical structures. Experimental validation—through in vitro tissue phantoms, ex vivo models, or in vivo animal studies—will be essential for confirming the accuracy of simulated outcomes and refining the integrated framework. In addition, exploring multiple coil designs, alignment scenarios, and WPT frequencies will help establish a more robust safety and performance envelope. Collectively, these directions will advance the development of fully optimized and clinically deployable implantable photonic systems.

## Figures and Tables

**Table 1:** Detailed results from the absorption spectra analysis.

Purpose	Poly Urethane	CSF	Gray, White Matter	Cerebellum
Diameter (mm)	0.8	0.5	2.5	3.5
Refractive index, real part	1.6	1.33	1.5	1.42
Absorption Coefficient (1/m)	0.0001	0.0013	0.0009	0.0002
Scattering Coefficient (1/m)	0.5	0.01	0.05	0.1
Conductivity (W/(m·k))	0.15	0.5	0.5	0.51
$\mathrm{Density}\;(kg/m^{3}$)	1200	1007	1045	1045
Heat Capacity (J/(kg·k)	1800	3850	3500	3653
Electrical Conductivity (S/m)	$1\times 10^{-5}$	0.12	0.12	0.45

**Table 2:** Wavelength-dependent absorption coefficients.

Wavelength (nm)	HbO_2_ (1/m)	Hb (1/m)	Wavelength (nm)	HbO_2_ (1/m)	Hb (1/m)
400	0.202300	0.128565	650	0.762966	0.424652
410	0.204633	0.143936	660	0.617055	0.349352
420	0.208964	0.165728	670	0.496848	0.286270
430	0.216663	0.195634	680	0.403002	0.235335
440	0.229764	0.235335	690	0.333382	0.195634
450	0.251071	0.286270	700	0.284202	0.165728
460	0.284202	0.349352	710	0.251071	0.143936
470	0.333382	0.424652	720	0.229761	0.128565
480	0.403002	0.511112	730	0.216663	0.118063
490	0.496848	0.606335	740	0.208903	0.111108
500	0.617055	0.70653	750	0.204633	0.106645
510	0.762966	0.806648	760	0.202301	0.10386
520	0.930128	0.900737	770	0.201097	0.102187
530	1.109794	0.982496	780	0.200503	0.101201
540	1.289221	1.045959	790	0.200221	0.100645
550	1.452905	1.086207	800	0.200093	0.100335
560	1.58467	1.1	810	0.200038	0.100169
570	1.67029	1.086207	820	0.200014	0.100083
580	1.7	1.045959	830	0.200005	0.100040
590	1.67029	0.982496	840	0.200001	0.100018
600	1.58467	0.900737	850	0.200002	0.100008
610	1.452905	0.806648	860	0.200002	0.100003
620	1.289223	0.70653	870	0.200007	0.100001
630	1.10979	0.606335	880	0.200002	0.100006
640	0.930128	0.511112	890	0.200007	0.100002

**Table 3:** Detailed results from the SAR analysis.

Purpose	Photonics	Head	Brain
SAR	$\frac{\sigma_{Photonics}\times {\left(|E\right|}^{2})}{\rho_{Photonics}}$	$\frac{\sigma_{Head}\times {\left(|E\right|}^{2})}{\rho_{Head}}$	$\frac{\sigma_{Brain}\times {\left(|E\right|}^{2})}{\rho_{Brain}}$
Electrical Conductivity, σ (S/m)	$1\times 10^{-5}$	0.15	0.12
$\mathrm{Density},\;\rho \;(kg/m^{3}$)	1300	1100	1050

**Table 4:** SAR values under optimized optical and electromagnetic parameters.

Model Type	Averaging Mass (g)	Average SAR (mW/kg)	Maximum SAR (mW/kg)
Head	10	7.4452 =(0.0074552 W/kg)	825.52 =(0.82552 W/kg)
Brain	10	9.3597 =(0.0093597 W/kg)	1037.8 =(1.0378 W/kg)

## Data Availability

The data that support the findings of this study are available from the corresponding authors upon reasonable request.
